# Directions Relative to the Prevention of and Treatment of Cholera, (Recently Issued by the Royal College of Physicians of London)

**Published:** 1849-01

**Authors:** John Ayrton Paris, Francis Hawkins

**Affiliations:** President; College of Physicians; Registrar; College of Physicians


### PATHOLOGY AND PRACTICE OF MEDICINE.

*Directions relative to the prevention of and treatment of Cholera, (recently issued by the Royal College of Physicians of London.)*—The Royal College of Physicians of London, feeling that, on the reappearance of epidemic cholera in England, the public may naturally look to them for advice and guidance, have deemed it proper to appoint a cholera committee, composed of physicians who hold important offices in the metropolitan hospitals, or who had extensive experience of the disease at its last visitation, to consider what measures it is expedient to adopt, with a view of preventing the spread of the disease, and of otherwise mitigating its evils.

The committee thus formed have, in compliance with the wish of the College, drawn up the following remarks and instructions, for the information of the public.

1st. Cholera appears to have been very rarely communicated by personal intercourse; and all attempts to stay its progress by cordons or quarantine have failed. From these circumstances, the committee, without expressing any positive opinion with respect to its contagious or non-contagious nature, agree in drawing this practical conclusion: that in a district where cholera prevails, no appreciable increase of danger is incurred by ministering to persons affected with it; and no safety afforded to the community by the isolation of the sick.

2d. The disease has almost invariably been most destructive in the dampest and filthiest parts of the towns it has visited. The committee would therefore urge on the public authorities the propriety of taking immediate steps to improve the state of sewers and drains; to cover those which are open; and to remove all collections of decaying vegetable and animal matter from the vicinity of dwellings. They would also impress on individuals, especially of the poorer classes, the great importance of well-airing their rooms, and of cleanliness in both their dwellings and persons.

3d. A state of debility or exhaustion, however produced, increases the liability to cholera. The committee therefore recommend all persons, during its prevalence, to live in the manner they have hitherto found most conducive to their health; avoiding intemperance of all kinds, and especially the intemperate use of ardent spirits and other intoxicating liquors. A sufficiency of nourishing food; warm clothing, and speedy change of damp garments; regular and sufficient sleep; and avoidance of excessive fatigue, of long fasting, and exposure to wet and cold, more particularly at night, are important means of promoting or maintaining good health, and thereby afford protection against the cholera.

The committee do not recommend that the public should abstain from the moderate use of well-cooked green vegetables, and of ripe or preserved fruits. A certain proportion of these articles of diet is, with most persons, necessary for the maintenance of health; and there is reason to fear, that if they be generally abstained from, now that the potato crop has in great measure failed, many persons, especially amongst the poor in large towns, will fall into that ill condition, which in its highest degree is known as scurvy, and that they will in consequence be the readier victims of cholera. The committee likewise think it not advisable to prohibit the use of pork or bacon; or of salted, dried, or smoked meat or fish; which have not been proved to exert any direct influence in causing this disease. Nothing promotes the spread of epidemic diseases so much as want of nourishment; and the poor will necessarily suffer this want, if they are led to abstain from those articles of food on which, from their comparative cheapness, they mainly depend for subsistence.

On the whole, the committee advise persons living in districts in which cholera prevails, to adhere to that plan of diet which they have generally found to agree with them; avoiding merely such articles of food as experience may have taught them to be likely to disorder the stomach and bowels.

4th. The committee are unable to recommend an uniform plan of treatment to be adopted by the public in all cases of looseness of the bowels, supposed to be premonitory of cholera. It is doubtless, very important that such ailments should be promptly attended to; but since they may arise from various causes, of which a medical man can alone judge, the committee deem it safer that persons affected with them should apply at once for medical assistance, than that they should indiscriminately use, of their own accord, or on the suggestion of unprofessional persons, powerful medicines, in large and frequently repeated doses. Should the looseness of the bowels be attended with feelings of great exhaustion and chilliness, the person should, of course, be placed in a warm bed, and the usual means of restoring warmth to the body be assiduously employed, until professional advice can be obtained.

5th. In order that the poor may have the means of obtaining such assistance promptly, the committee recommend that the proper authorities should at once establish dispensaries in those parts of the town which are remote from the existing medical institutions; and that they should also take steps to provide distinct cholera hospitals, which it will require some time to organize, and which they believe will be found to be absolutely necessary, should the epidemic prevail in this metropolis with a severity at all approaching that which it manifested on its first appearance in England. The committee wish it to be clearly understood, that they do not recommend the establishment of such cholera hospitals, on the ground of effecting the separation of the sick from the healthy, and of thus preventing the spread of the disease; but solely in order that, should the epidemic prove severe, proper attendance and prompt treatment may be ensured for the sufferers from cholera among the poorest and most destitute class. The existing hospitals, even if the authorities should consent to the admission of persons ill of cholera, could not furnish the requisite accommodation, unless they were shut against persons labouring under other severe diseases—a measure which, at the approach of winter especially, would add much to the distress of the poor.

6th. In conclusion, the committee would urge on the rich, who have comparatively little to fear for themselves, the great duty of generously and actively ministering to the relief of the poor, while the epidemic prevails; bearing in mind that fuel, and warm clothing, and sufficient nourishment, are powerful safeguards against the disease.

They deem it most desirable that the parish authorities should at once improve the diet, and increase the comforts, of the poor under their charge; and that the wealthy should form societies for the supply of food, clothing, and fuel, to those who, though not paupers, still need charitable assistance in the present emergency.

Such measures, which it is the duty of those possessed of power and wealth to adopt, would, the committee believe, if liberally carried out, deprive the cholera of half its victims.

JOHN AYRTON PARIS, President.
FRANCIS HAWKINS, Registrar.

*College of Physicians, Oct.* 28, 1848.

[*London Lancet.*

